# Identification and classification of small molecule kinases: insights into substrate recognition and specificity

**DOI:** 10.1186/s12862-015-0576-x

**Published:** 2016-01-06

**Authors:** Krishnadev Oruganty, Eric E. Talevich, Andrew F. Neuwald, Natarajan Kannan

**Affiliations:** Department of Biochemistry & Molecular Biology, University of Georgia, Athens, GA 30602 USA; Department of Pathology and Helen Diller Family Comprehensive Cancer Center, University of California, San Francisco, CA 94158 USA; Institute for Genome Sciences and Department of Biochemistry & Molecular Biology, School of Medicine, University of Maryland, Baltimore, MD 21201 USA; Institute of Bioinformatics, University of Georgia, Athens, GA 30602 USA

**Keywords:** Kinase superfamily, Aminoglycoside kinase, Antibiotic resistance, Substrate specificity, Enzyme evolution

## Abstract

**Background:**

Many prokaryotic kinases that phosphorylate small molecule substrates, such as antibiotics, lipids and sugars, are evolutionarily related to Eukaryotic Protein Kinases (EPKs). These Eukaryotic-Like Kinases (ELKs) share the same overall structural fold as EPKs, but differ in their modes of regulation, substrate recognition and specificity—the sequence and structural determinants of which are poorly understood.

**Results:**

To better understand the basis for ELK specificity, we applied a Bayesian classification procedure designed to identify sequence determinants responsible for functional divergence. This reveals that a large and diverse family of aminoglycoside kinases, characterized members of which are involved in antibiotic resistance, fall into major sub-groups based on differences in putative substrate recognition motifs. Aminoglycoside kinase substrate specificity follows simple rules of alternating hydroxyl and amino groups that is strongly correlated with variations at the DFG + 1 position.

**Conclusions:**

Substrate specificity determining features in small molecule kinases are mostly confined to the catalytic core and can be identified based on quantitative sequence and crystal structure comparisons.

**Electronic supplementary material:**

The online version of this article (doi:10.1186/s12862-015-0576-x) contains supplementary material, which is available to authorized users.

## Background

Eukaryotic-Like Kinases (ELKs) phosphorylate small metabolites such as choline, aminoglycoside and fructosamine [1, 2]. ELKs are evolutionarily related to Eukaryotic Protein Kinases (EPKs) that regulate diverse cellular processes through the controlled phosphorylation of serine, threonine and tyrosine residues on protein substrates [3–7]. The EPK and ELK catalytic domains share a bi-lobal structure consisting of an N-terminal ATP binding lobe and C-terminal substrate binding lobe [8–11]. While the ATP binding lobe is similar in EPKs and ELKs, the substrate binding lobe differs, presumably due to the nature of substrates that EPKs and ELKs phosphorylate [1, 2]. Crystal structures of EPKs bound to peptide substrates have provided insights into substrate recognition and specificity [11, 12]. Likewise, peptide library based assays have revealed short sequence motifs that act as high affinity kinase substrates [13–15]. The linear peptide motifs have also been mapped to (non-linear) structure based recognition motifs to detect full-length protein substrates in vivo [16]. More recently, a sparse network of residues in the protein kinase domain has been suggested to contribute to substrate specificity [17], though for most kinases, domains and sequences outside the kinase domain play a major role in substrate recognition [18]. For instance: docking site interactions govern substrate recognition in MAPKs [19, 20]; the SH2-SH3 domain affects substrate specificity in some tyrosine kinases [21, 22]; and scaffolding proteins provide substrate specificity in many EPKs [23–25].

Although catalytic activity and substrate recognition in many EPKs are controlled by phosphorylation-mediated conformational changes in the protein kinase domain, most ELKs are constitutively active single domain proteins with little or no post-translational regulation. Furthermore, unlike EPKs, ELK substrate specificity determinants are confined to the catalytic core, at least in those ELKs for which substrate bound crystal structures are available. This provides an opportunity to investigate the relationships connecting sequence, structure and substrate specificity in ELKs through quantitative comparisons of existing sequences and crystal structures. The study of ELKs is gaining importance due to rise of antibiotic resistance, where aminoglycoside kinases/phosphotransferases (APH, a family of ELKs) play a major role [26–28]. Previous structure-guided approaches have helped identify small molecule inhibitors that can reverse antibiotic resistance in a sub-class of aminoglycoside kinases [29]. The choline kinases, another class of ELKs, have emerged as attractive targets for cancer chemotherapy [30, 31]. Thus, a deeper understanding of the relationships connecting sequence, structure, function and evolution in ELKs can aid in the design of selective inhibitors.

Early on, sensitive sequence comparison methods enabled the identification and classification of ELK sequences in eukaryotic and prokaryotic genomes. Koonin et al*.* used paralog detection and PSI-BLAST searches to discover novel ELK families [32]. Likewise, Krupa and Srinivasan, using sequence-profile alignment methods, identified novel lipid kinases that are distantly related to protein kinases [33]. A motif based metagenomic survey allowed Kannan et al. to broadly classify ELK sequences into major groups and families and identify novel families such as maltose kinase and bacterial spore kinases [1] that have subsequently been validated through structural studies [34, 35].

Although some ELK crystal structures are available, they are still far underrepresented in comparison to EPKs. Nevertheless, the availability of ELK structures from major groups has enabled structure-based classification of the EPK/ELK superfamily. Bourne and Scheef generated a structure-based phylogeny of the EPK/ELK superfamily using structure-based sequence alignment methods [36]. They found that choline kinases and aminoglycoside kinases are not closely related, but could not resolve the deeper evolutionary relationships due to the lack of structural information. At a much deeper level, other groups analyzed the structural evolution of the protein kinase-like superfamily in comparison to other ATP binding proteins and found that protein kinases show greatest structural similarity to ATP grasp proteins, suggesting descent from an ATP grasp-like domain [37, 38]. However, despite these studies and the exponential growth of ELK sequences in sequence databases, the sequence and structural determinants of ELKs functional specificity have not been systematically explored. One of the major hurdles in such an analysis is the presence of long inserts within the kinase domain, which hinders large-scale quantitative comparisons of ELK sequences and crystal structures.

In this study, we use a profile based sequence alignment program with manually curated structural alignments to provide an accurate alignment of all ELK sequences. We develop a classification of ELKs based on sequence divergence of key motifs in the kinase domain. We define the common minimum core domain that is present in all members of this superfamily. An analysis of discriminating sequence patterns within this ELK core domain reveals that small molecule kinases fall into distinct subgroups, several of which are defined for the first time here. A phylogenetic analysis suggests that, with the exception of the APH(2”) and APH(3’) enzyme families of aminoglycoside kinases, these groups are monophyletic. Structural and Bayesian analysis of those conserved residues that best discriminate between subgroups suggests a simple rule for substrate specificity in APH(2’) and APH(3’) enzymes. We have also discovered examples of unique residue patterns that determine the ATP orientation required for substrate phosphorylation in different ELKs. The definition of unique patterns of amino acids in each group provides a rational basis for the classification of existing small molecule groups and provides a basis for prediction of substrate binding resides in novel ELKs. Finally, this study of ELKs provides a framework within which substrate specificity and regulation across all kinases may be further investigated.

## Results and Discussion

**Fig. 1:**
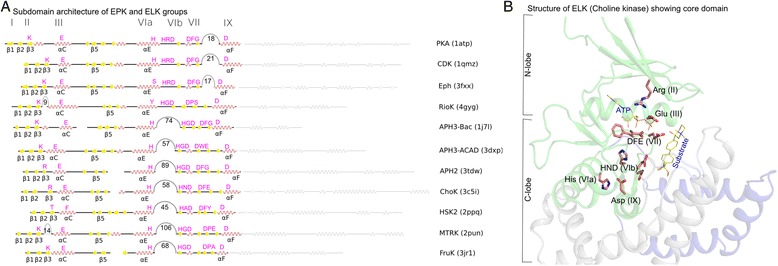
The kinase core domain in a representative set of EPKs and ELKs. **a**. Secondary structure labels are indicated below the sequence, and Hanks and Hunter subdomain notations are shown above the sequence. Insert segments longer than 5 residues are indicated as arcs in the sequence, and the numbers indicate the average insert size. **b**. Core domain highlighted in the crystal structure of choline kinase

A core domain commonly shared by EPKs and ELKs was defined based on available sequences and crystal structures (see Methods and Fig. 1). The core domain encompasses the ATP and substrate binding lobes of the kinase domain, namely sub-domains I-V of the N-terminal ATP binding lobe and sub-domains VIa, VIb, VII and IX of the substrate binding lobe (Fig. 1). ELKs generally have two segments outside of the core domain. One is an insert between the E-helix (subdomain VIa) and catalytic loop (subdomain VIb) that is absent in most EPKs. Most ELKs also contain a C-terminal helical subdomain directly following subdomain IX, which EPKs lack. As noted previously [39], the exaggerated activation segment connecting the DFG motif and F-helix to the C-terminal G-, H- and I-helices is unique to EPKs and contributes to protein-substrate binding. Apart from these major EPK- and ELK-specific insert segments, a few ELK groups show additional inserts within the core domain. For instance Kdo, Rio, MTRK (methylthioribose kinase), and UbiB contain an insert between β-sheet 3 in the N-lobe (subdomain II) and the C-helix (subdomain III). UbiB also contains a 70–90 residue insert in the region corresponding to the activation loop in EPKs, but the function of this insert in UbiB is unknown. From the core domain alignment, we constructed a hierarchical set of sequence profiles representing major EPK and ELK groups and the families/sub-families within each group.

### Phylogenetic analysis of core kinase domain delineates monophyletic ELK groups

**Fig. 2:**
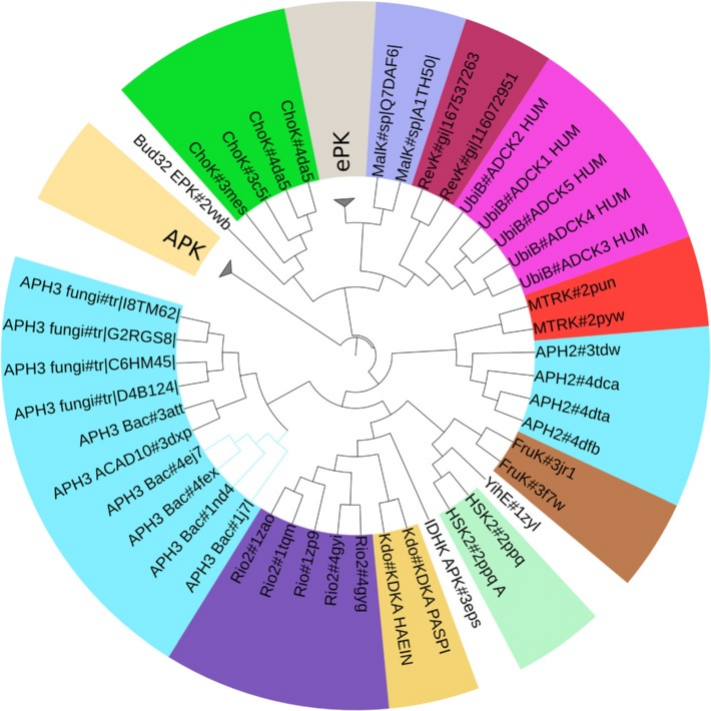
A schematic cladogram showing the relationships between different ELK and EPK groups with APKs serving as an outgroup. The full tree is given as figure 2a. The proteins shaded with the same color are currently considered part of the same ELK group. The APH2 and APH3 groups cluster separately, with APH3 showing further subdivisions as indicated in the figure. Wherever possible sequences of known structure are included and the PDB ids are indicated next to the protein name in the cladogram

Sequence similarity based clustering of ELK and EPK core domain sequences revealed 758 clusters at 60 % sequence identity with 408 EPK clusters and 250 ELK clusters. The consensus of each cluster and representative sequences of known structure were used to derive maximum likelihood trees. The phylogenetic analysis described here refers to the maximum likelihood tree with representative sequences (Fig. 2).

Since the phylogenetic tree was generated based on the core domain alignment (i.e., excluding EPK and ELK specific inserts), we wanted to determine whether the core domain contains sufficient information to recapitulate known evolutionarily relationships in EPKs and ELKs. As a test, we compared the maximum likelihood tree of all EPKs generated based on the core domain to one based on the full-length kinase domain [40]. The core domain based tree (Additional file 1: Figure S1A) captures known evolutionary relationships by correctly clustering related kinases similarly to the full-length domain tree [40]. As an additional test, we also generated a maximum likelihood tree of the Phosphoinositide-3 kinases (PI3Ks) [41], a class of atypical kinases (APKs) distantly related to EPKs and ELKs, based on the commonly shared core domain. As expected, we see partitioning of inositol phosphorylating and protein phosphorylating PI3Ks [42] based on the core PI3K alignment. Thus, the core domain encompassing subdomains I–VII and IX possesses sufficient evolutionary information to correctly classify EPKs, and distantly related APKs when analyzed individually.

#### ELK re-classification reveals distinct APH subgroups

We performed core-domain-based maximum likelihood phylogenetic analysis of APKs, EPKs and ELKs using representative sequences that, wherever possible, corresponded to proteins of known structure. PI3K and other APKs such as Fam20C [43] were used as outgroups, and the tree was rooted at the branch point of the APK groups (Fig. 2). The tree shows that EPKs generally cluster together and that ELKs diverge from currently existing classifications. The full tree (Additional file 2: Figure S2A) additionally suggests that the pknB group, which consists of bacterial protein kinases, is more closely related to EPKs than to ELKs. The subdomain architecture of the full-length pknBs is also closer to EPKs than to ELKs [44].

The maximum likelihood tree bootstrap values (from 100 alternate trees) (Additional file 2: Figure S2A) suggest that nodes separating groups with high sequence identity have high confidence values. Also, for known homologous groups with approximately 20 % sequence identity such as the Rio kinase and Kdo kinases, the branch point has a bootstrap value of 68 %, supporting the evolutionary relationship. The bootstrap values for branch points between other ELK groups are generally well below 50 %, suggesting that core domain divergence (at least with current methods) cannot be used to determine unambiguously the deeper evolutionary history between divergent ELK groups.

*Two distinct sub-groups of bacterial APHs*: The APH group, which is typically classified as a single enzyme family, shows the highest divergence within the core domain. Two distinct clusters can be discerned from the tree, and the clusters are defined as the APH3 group and APH2 group based on annotations of a few proteins in each cluster. The naming reflects the fact that APH3 group enzymes phosphorylate kanamycin at the 3’ position whereas the APH2 group enzymes phosphorylate kanamycin at the 2” position. The APH3 family shows distinct sub-clusters depending on its occurrence in eukaryotes and prokaryotes (Additional file 2: Figure S2B). The eukaryote APH3 sequences can be further divided into two broad subclasses: One of these, the APH3_ACAD subclass, occurs in nearly all eukaryotes and is associated with acyl-CoA-dehydrogenase enzymes (e.g., ACD10_HUMAN) (representative pdbid: 3dxp in Fig. 2). Another APH family, which appears to be fungi-specific, is related to APH3_ACAD; it is named APH3_Fungi (branch point bootstrap value 75 %). The only well characterized APH3 enzymes are from bacteria (APH3_Bac); these cluster together (branch bootstrap values >90 %), except for a mycobacterial enzyme that clusters with APH3_ACAD and that was initially classified as APH3_Bac based on organism distribution (pdbid: 3att). Thus our analysis suggests a family of bacterial APH3 enzymes with an as-yet-unknown function. The structure of mycobacterial APH3_Bac (pdbid: 3att) shows that the enzyme adopts the kinase fold with unusually long β-strands in the N-lobe.

APH2 and APH3-Bac tertiary structures adopt slightly different conformations (Additional file 3: Figure S3). APH2 enzymes have a shorter F-helix (subdomain IX) and a shorter G-rich loop (Additional file 3: Figure S3B) but possess a longer substrate binding C-tail (Fig. 1), which adopts a unique conformation in each case, as does the substrate binding ELK-specific-insert (Additional file 3: Figure S3A). Apart from the major split in the APH3 family, the phylogenetic tree in Fig. 2 suggests that each ELK group is monophyletic. A multiple category Bayesian partitioning with pattern selection (mcBPPS) sampler was used to find sequence patterns distinctive of each ELK group—as described in the following sections. In each case, an ELK group was compared against an alignment of all other ELK groups. For the sake of brevity, we do not discuss (previously noted [1, 39]) conserved catalytic residues shared by EPKs and ELKs (see Fig. 1), such as the magnesium binding aspartate (the DFG-Asp in EPKs) and the catalytic aspartate (the HRD-Asp in EPKs).

**Fig. 3:**
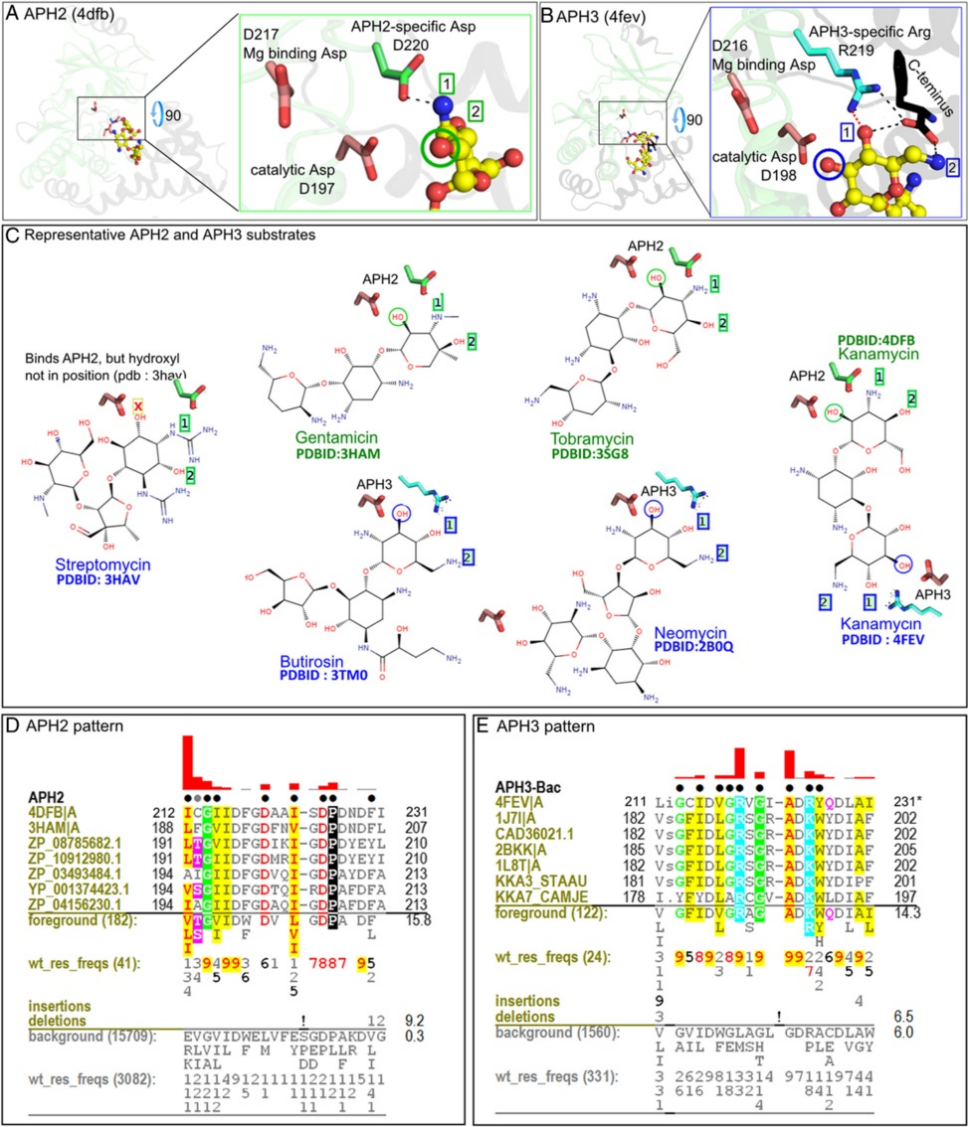
The simple substrate recognition rule revealed through mcBPPS analysis. The residues shown as APH2- or APH3-specific are shown in the respective structure figure panels. **a** APH2 catalytic site showing the unique residues (carbon atoms colored green) and catalytic aspartates (carbon atoms colored light pink). The receiving hydroxyl group is circled in red. **b** APH3 catalytic site showing the unique residues (carbon atoms colored blue) and catalytic residues. **c** Several APH2 and APH3 substrate bound conformations present in PDB are shown schematically. The substrate hydroxyl is circled in red in each case. For each APH2 substrate, a schematic catalytic aspartate (colored light pink) and APH2-speciifc aspartate (colored blue) are shown that provide the substrate binding specificity. For each APH3 substrate, the catalytic aspartate and APH3-conserved arginine are shown schematically as binding to a specific pattern of chemical groups on the substrate. For APH2 substrates, starting from the substrate hydroxyl, the OH-NH2-OH pattern is shown, whereas for APH3, the OH-OH-NH2 pattern is shown. The schematic of PDB structure 3HAV shows that when an APH2 enzyme is presented with APH3 substrate (streptomycin), the substrate still binds in an APH2 recognition pattern (OH-NH2-OH) but without correct stereochemistry of the substrate hydroxyl for substrate phosphorylation to take place. **d** mcBPPS output showing flanking segments of the DFG motif in a Contrast Hierarchical Alignment (CHA). The CHA shows representative APH2 sequences as the display alignment, all APH2 sequences as foreground alignment (182 sequences) and all ELK sequences as background alignment (15,790 sequences). The foreground and background alignment are shown as residue frequencies below the display alignment. Residue frequencies at each aligned position are given in integer tenths; for example, an ‘8’ indicates that 80–90 % of the sequences in the foreground alignment match the corresponding pattern residue (with ‘!’ indicating 100 %). The first of these ‘residue frequency’ lines reports the virtual number of aligned sequences after down-weighting for redundancy. Directly below this are shown the number of insertions and deletions at each position, again in integer tenths. The black dots above the alignment indicate the pattern positions that were identified by the mcBPPS sampler and which were used to classify the APH2 sequences. To enhance interpretation of the alignment, pattern-matching residues are colored, with biochemically similar residues colored similarly. For example, acidic residues are shown in red, basic residue in cyan and hydrophobic residues in yellow; histidine, glycine and proline are each assigned a unique color. The height of the red bars above the alignment quantify (using a semi-logarithmic scale) the degree to which residue frequencies in the foreground diverge from the corresponding positions in the background at each position. **e** CHA alignment showing representative bacterial APH3 sequences as display, all bacterial APH3 sequences as foreground (122 sequences) and APH3-ACAD sequences as background (1560 sequences)

*Sequence signatures reflect substrate specificity within the APH family:* Our mcBPPS analysis revealed that the magnesium binding loop in subdomain VII of APH2 is characterized by an APH2-specific aspartate (D) right after the DFG motif (DFGD). In the crystal structures of APH2 bound to kanamycin (pdbid : 4dfb) or to tobramycin (pdbid : 3sg8) (Fig. 3a, c) this aspartate hydrogen bonds to an amide in the aminoglycoside moiety. In all APH2 structures with bound aminoglycoside, we observe that the phosphorylatable hydroxyl (circled in green in Fig. 3) is adjacent to an amide group (labeled 1), which is held in place by the APH2-specific aspartate (D220 in Fig. 3a). In contrast, in APH3 enzyme structures, the phosphorylatable hydroxyl is adjacent to another hydroxyl group that is stabilized by an APH3-specific arginine (DFGR motif, R219 in Fig. 3b). APH3 enzymes have an unusually short C-tail, the terminal residue of which (F271 in pdbid 4fev) is also stabilized by a hydrogen bond with this APH3 arginine (R219). The C-terminal residue also hydrogen bonds to an amide group located 2 carbon atoms away from the phosphorylatable hydroxyl.

The substrate specificity in APH2 and APH3 enzymes is currently poorly understood. Based on our analysis, we predict that residues at the DFG + 1 position contribute to APH2 versus APH3 substrate specificity. Within the terminal glycoside, APH2 prefers OH-NH2-OH ordered carbon atoms whereas APH3 prefers OH-OH-NH2 ordered carbon atoms (see Fig. 3 for details). The importance of this simple rule can be gauged by two observations. First, both APH2 and APH3 enzymes phosphorylate kanamycin, but they bind kanamycin in different orientations. Second, the structure of APH2 bound to streptomycin (an APH3 substrate) leads to a non-productive complex with binding orientation similar to APH2. Based on these rules, hygromycin B, which has both APH2 and APH3 type motifs should be phosphorylated at the 6’ position by an APH2 enzyme and, indeed, hygromycin B kinase (KHYB_STRHY) is an APH2 enzyme conserving an aspartate at the DFG + 1 position (DFTD motif). Another enzyme from *E.coli* (KHYB_ECOLX) that phosphorylates hygromycin B at an APH3 type motif conserves an arginine at the DFG + 1 position (DNGR motif).

### Sequence signatures of ELKs

We next determined whether residues distinguishing major ELK groups likewise correlate with substrate specificity. Core residues characteristic of major ELK groups was identified using mcBPPS (see methods) and non-core residues were identified using an alignment of full-length sequences. In the subsequent subsections, we discuss core and non-core residues associated with substrate recognition and specificity.

#### Core domain evolved to recognize diverse substrates in ELKs

**Fig. 4:**
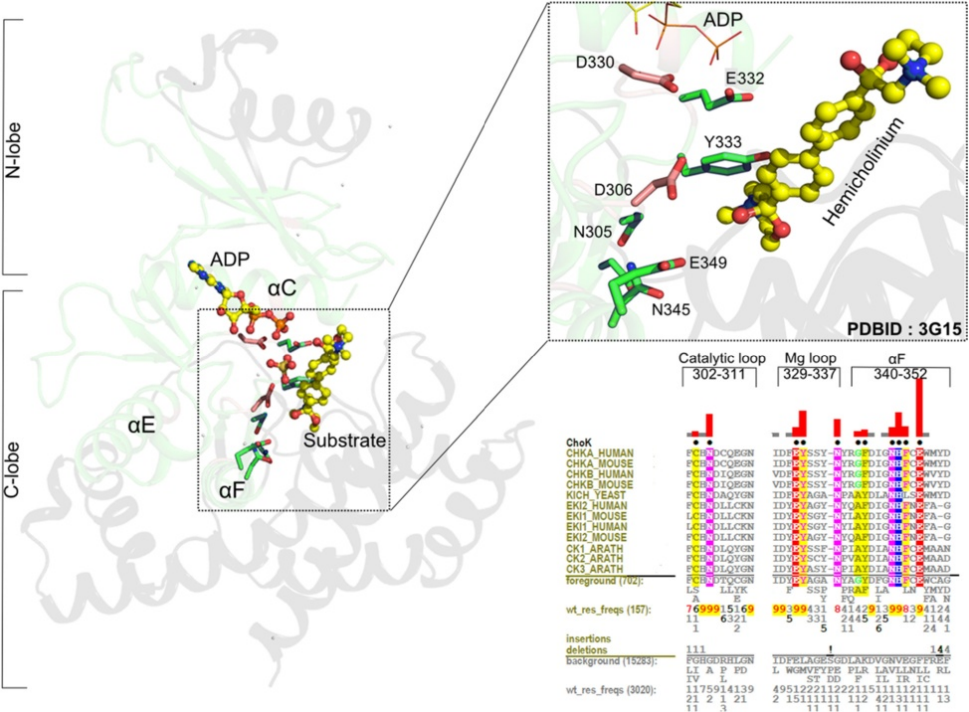
The substrate recognition region in Choline kinases. On the left is shown the structural context of the substrate binding region. The inset shows conserved residues within the substrate binding region that are most distinctive of these kinases based on mcBPPS analysis. In the structure figures, the green region corresponds to the core domain whereas the black regions are outside of the core. Residues that are Choline kinase-specific are shown with green carbon atoms and catalytic residues are shown in light pink carbon atoms. The substrate analog hemicolinium is shown in yellow CPK representation. A Contrast Hierarchical Alignment (CHA) in the bottom right panel shows the constraints imposed on residues in key regions of the kinase domain. CHA shows representative Choline kinase sequences as the display alignment; all Choline kinase sequences (702 sequences) as foreground and other ELK sequences (15283 sequences) as background. CHA coloring scheme and representation is similar to that described in Fig. 3d

*ChoK–specific residues assist in substrate binding and catalysis:* Choline and ethanolamine kinases play a major role in eukaryotic membrane maintenance and catalyze the first committed step in the Kennedy pathway for phosphatidylcholine synthesis. Some of the most distinguishing choline kinase residues/motifs as revealed by mcBPPS analysis are shown in Fig. 4. Notably, many of the choline kinase-specific residues lie near the bound hemicholinium (a choline substrate analog) and either directly or indirectly interact with the substrate. The most distinctive residue is an invariant glutamate in subdomain IX (F-helix) that binds the positively charged substrate. *In vitro* experiments on *C.elegans* choline kinase A2 show that mutation of this glutamate to an alanine (E320A) increases *K*_*m*_ for choline 3 fold without an appreciable change in *k*_*cat*_ [45], indicating a role in substrate binding but not catalysis. Other ChoK-specific residues shown in Fig. 4 are involved in catalysis. For example, mutation of the glutamate within the magnesium binding loop (DFE-Glu, E332) to an aspartate reduces *k*_*cat*_ by half and increases Km for choline 3 fold. Mutation of the glutamate to an alanine reduces *k*_*cat*_ 10 fold and increases choline *K*_*m*_ 10 fold [45]. This suggests that E332 plays a role in both substrate binding and catalysis. Two of these residues, an asparagine in the catalytic loop (N305) and an asparagine in the C-terminal tail (N345), are also distinctive of choline kinases. Mutation of the asparagine to an alanine drastically reduces *k*_*cat*_ and slightly increases *K*_*m*_ for choline [35]. The two asparagines form a bridging interaction between the active site and the substrate binding inserts. Presumably, the integrity of the substrate binding site is lost when these residues are mutated leading to lower affinity for substrate. Thus, the distinguishing choline kinase-specific residues contribute to substrate binding and catalysis.

##### MTRK signature sequences line substrate binding regions

**Fig. 5:**
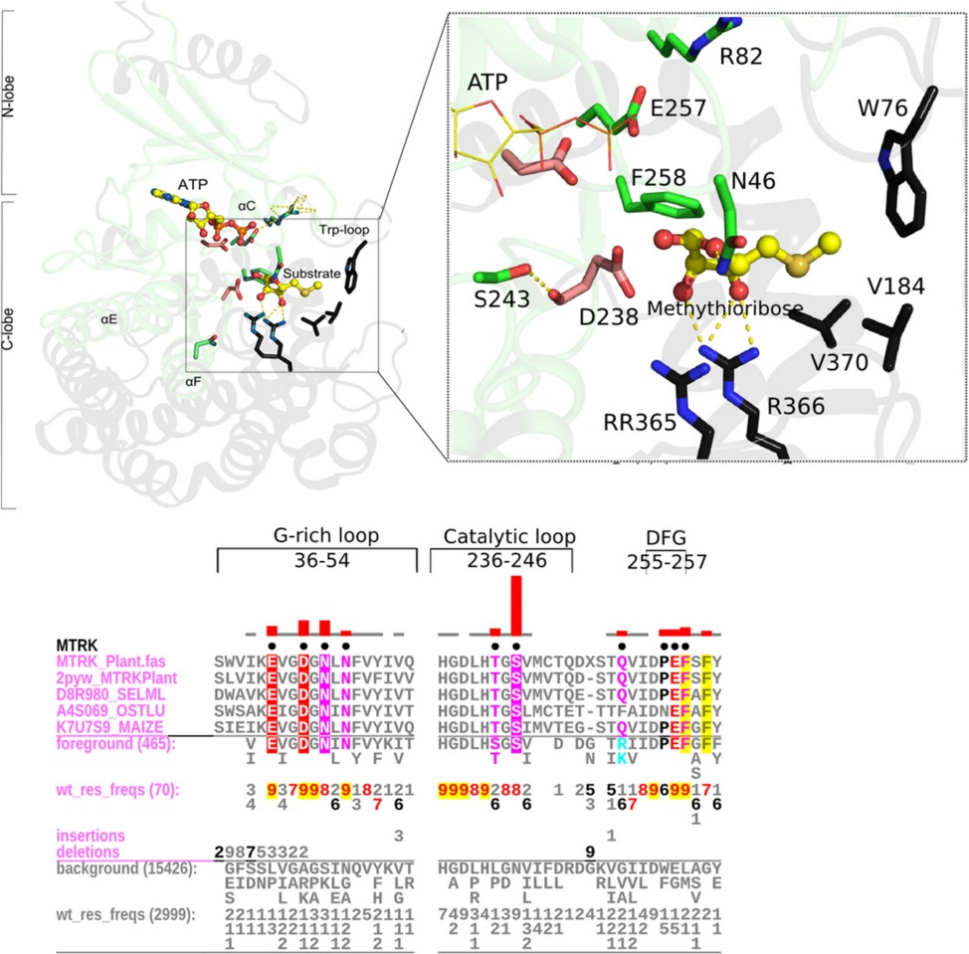
The substrate recognition region in MTRK. The top panel shows the structural context and an inset showing the details of the substrate binding region in a plant MTRK. In the structure figures, the green regions correspond to the core domain whereas the black regions are outside of the core. Residues that are MTRK specific are shown with green carbon atoms and catalytic residues are shown in light pink carbon atoms. Important residues in the non-core region that bind substrate are shown in black. A non-core insert part of the substrate binding ‘trp-loop’ (containing W76) is also shown in context of the substrate. The mcBPPS pattern characteristic of MTRK is shown using CHA. CHA shows representative bacterial MTRKs as the display alignment, all MTRK sequences (465 sequences) as the foreground alignment and other ELK sequences (15,426) as the background. CHA coloring scheme and representation is similar to that described in Fig. 3d

MTRKs are metabolic enzymes that phosphorylate methylthioribose—an essential step in the methionine salvage pathway in bacteria and plants. MTRK-specific residues (Fig. 5) are present both in the N-lobe and C-lobe of the kinase domain and often coordinate directly or indirectly with the substrate. One of the most distinctive MTRK-specific residues is a serine (S243) in the catalytic loop that replaces the Mg^2+^ coordinating asparagine (N171^PKA^). In the crystal structure of plant MTRK bound to substrate (PDB: 2PYM), S243 hydrogen bonds to the backbone of the catalytic aspartate (D238), which coordinates with the methythioribose substrate. Other MTRK-conserved residues likewise contribute to the unique modes of ATP binding and substrate recognition [46]. For example, E257 in the DPE motif (DFG motif in EPKs) coordinates with MgATP, and the MTRK-conserved phenylalanine at the DFG + 1 position (F258 in Fig. 5) is part of a hydrophobic pocket that binds the methylthio group of the substrate [47]. The methylthio group is also bound by a conserved tryptophan in the so called trp-loop, which is a MTRK-specific insert between β-strand 3 and the C-helix. The insert residues hydrogen bond with an MTRK-specific arginine in the C-helix (R82), which helps position the substrate binding loop. Other MTRK conserved residues in the G-rich loop and F-helix, likewise, contribute to substrate recognition by positioning substrate binding motifs, such as the twin-arginine motif, which contributes to substrate recognition [47]. We propose that strong selective pressures are imposed on these residues due to their roles in substrate recognition and specificity.

##### Other ELK group-specific residues determine substrate binding specificity

**Fig. 6:**
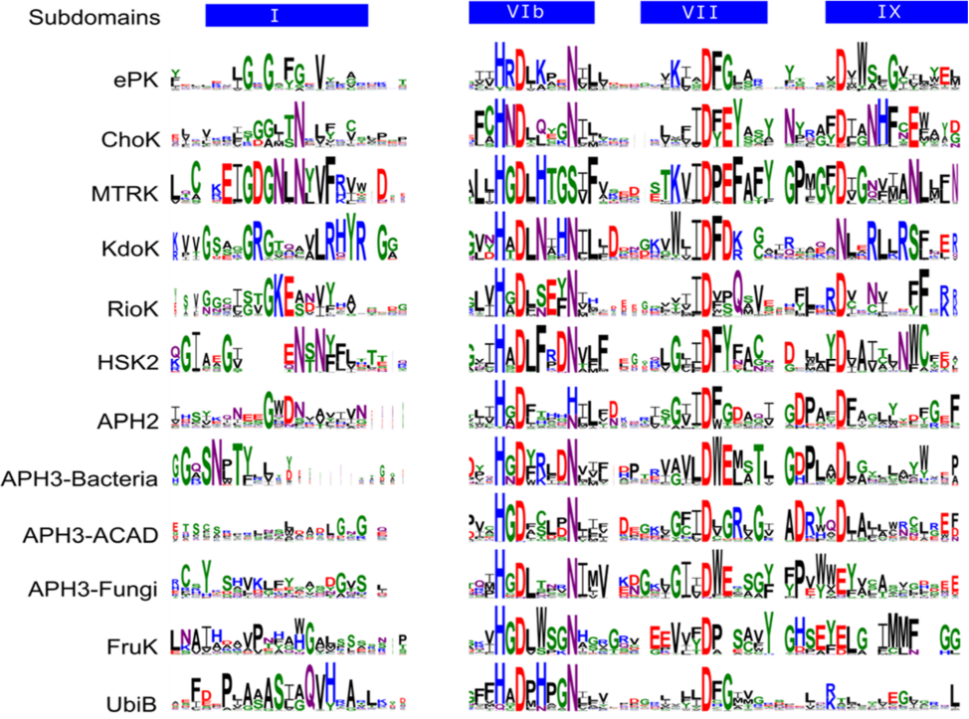
Weblogos showing EPK and ELK conserved motifs in key functional regions of the kinase domain. The GxGxxG motif in EPKs (Sub-domain I) shows the highest divergence in ELKs. Conserved motifs have the highest information content as indicated by the size of the letters

Examination of group-specific residues in other ELKs reveals a common trend wherein regions involved in substrate recognition are under selective pressure. These regions are summarized in Fig. 6, and include the catalytic loop, magnesium binding loop and the F-helix region. In this section, these regions are analyzed in other ELK groups to gain insight into substrate specificity. We also present guidelines for predicting substrate-binding residues.

Kdo kinases are closest to the canonical core domain defined in this work, as they show very few inserts within or outside of the common core. In vivo mutagenesis studies have shown that Kdo kinase is active in catalyzing the phosphorylation of Kdo (3-deoxy-D-manno-octulosonic acid) at the O-4 position in *H.influenzae*. As for the MTRK and Choline kinases, group-specific residues are found in the F-helix, and the magnesium binding and catalytic loops (Fig. 6). A model of Kdo based on a Rio kinase structure (Additional file 4: Figure S4) suggests that the magnesium binding loop lysine (subdomain VII) and the two arginines in the F-helix (subdomain IX) are juxtaposed for hydrogen bonding with the substrate. The twin-arginine type motif is similar to that seen in MTRK and could help orient the sugar moiety. Given that the substrate sugar moiety in Kdo is larger than that of ribufuranose bound to MTRK, additional hydrogen bonding residues may be needed to orient it optimally for catalysis. A similar concentration of charged groups around Kdo moieties is seen in Kdo synthetases (pdbid 3k8d) [48] that are not related to protein kinases, suggesting convergent evolution of Kdo binding pockets within distinct folds.

Apart from residues in the substrate binding lobe, the catalytic loop (subdomain VIb) in the ATP binding lobe also shows unique patterns in each ELK group. When compared to EPKs, these patterns suggest that apart from catalytic residues, the catalytic loop could also play a role in substrate binding or help in facilitating the release of leaving groups. For instance, all EPKs have an arginine/lysine (K168^PKA^) in the catalytic loop that has been suggested to stabilize reaction intermediates [49–51]. ELKs generally lack such an arginine or lysine residue. The only exception is HSK2 (Homoserine kinase), which phosphorylates amino acid hydroxyl groups. Hence conserved non-catalytic residues may help discriminate between different substrates. Similarly, some APH enzymes conserve an arginine in the catalytic loop [52], and kanamycin kinase has been shown to phosphorylate peptides on serine residues [53]. PI3K conserves an arginine or histidine in the catalytic loop and phosphorylates proteins such as mTOR and ATR/ATM kinases. Although the APK, AlphaK, lacks an arginine or lysine in its catalytic loop, a distal segment of AlphaK conserves an arginine that structurally corresponds to the EPK catalytic loop arginine/lysine (Additional file 5: Figure S5). The convergent evolution of catalytic loop arginine/lysine residues in AlphaK suggests that they play a fundamental role in catalysis. The conservation of histidine in UbiB kinases, which is a basic amino acid as are lysine and arginine, suggests that these kinases may possess peptide phosphorylation activity. ADCK3, a member of the UbiB family, exhibits autophosphorylation activity [54], suggesting that it may phosphorylate protein substrates. Thus, it seems likely that the presence of arginine or lysine near the substrate is required for efficient phosphorylation of hydroxyl groups on amino acids.

#### Diverse ATP binding and catalytic regions

Many of the group-specific residues are conserved in the N-lobe region surrounding the ATP binding site. For instance, APH2 conserves a glutamate near the G-rich loop. The conformation of the ATP binding site is slightly different in each ELK (Additional file 6: Figure S6). APH2 enzymes prefer GTP over ATP in the active site. [55]. The ATP binding region in some ELK groups, such as HSK2, lack certain residues that are invariant in other ELKs and EPKs. These residues (the β3-lysine, K72^PKA^, and the C-helix-glutamate, E91^PKA^) are simultaneously lost suggesting a different mode of ATP binding, or perhaps a different metal cofactor dependence, for these enzymes. The ATP binding region of Rio kinases is unique in that serine residues replace glycine residues within the G-rich loop. The UbiB group likewise conserves a distinctive A-rich loop in place of the G-rich loop and, for ADCK3, mutation of these alanines to glycines confers the ability to autophosphorylate [54]. The orientation of ATP in the binding pocket differs between each of five representative ELK groups (Additional file 6: Figure S6). Hence each ELK group appears to bind and orient ATP and substrate uniquely, perhaps to provide an optimal environment for phosphate transfer.

## Concluding Remarks

Prediction of protein kinase substrates is an important unsolved problem because of the transient nature of kinase substrate interactions and the role of scaffolding proteins and localization in substrate specificity [18]. In contrast, for ELKs, the specificity determining features appear to be confined to the catalytic core. This provides an opportunity to predict substrate determining features based on quantitative comparison of ELK sequences and crystal structures. Analysis of discriminating patterns in various ELK groups reveals key residues and structural motifs associated with substrate specificity. Unique residue patterns in each ELK group not discussed in this study may be involved in conserved protein-protein interactions or regulatory functions that are currently unknown.

The Rio/Kdo kinases are structurally most similar to the core domain, raising the possibility that they most closely resemble the common ancestor of all kinases in this superfamily. The substrate-binding and regulatory properties of extant ELKs are due to co-evolution of additional insert regions with core domain variations. Such co-evolution is illustrated by Kdo and MTRK, both of which show two arginine residues that, based on available structural data, bind sugar substrates (Fig. 5 and Additional file 4: Figure S4). In case of Kdo, the twin arginines are part of the core domain, but in MTRK, they are in the substrate binding insert, which is held in place by MTRK-specific residues in the C-helix. EPKs similarly evolved substrate binding segments outside the core domain, namely the activation loop and G-, H- and I- helices; these are held in place by the HRD-arginine and an F-helix-tryptophan, both of which are EPK consensus residues in the core domain. This suggests that kinase substrate specificity has evolved in a modular fashion with anchoring residues in the core domain co-evolving with substrate binding segments.

Our studies also provide a new classification scheme for APH enzymes based on differences in the core domain, which indicate two distinct clades of APH enzymes: an APH2 clade exclusive to bacteria and an APH3 clade present in both eukaryotes and prokaryotes. The divergence in substrate binding regions provides a rational basis for classification of APH groups. Examination of unique patterns revealed a hitherto unappreciated substrate selectivity principle in APH2 and APH3 (an OH-OH-NH2 pattern in APH2 and an OH-NH2 pattern in APH3; Fig. 3). This principle informs the prediction of substrates and the design of antibiotics that cannot be inactivated by these enzymes. Metabolic enzymes such as N-acetyl glucose kinase (NahK; pdbid : 4ocv [56]) may have been the ancestral form of APH enzymes: The rigid and small pocket in NahK, which binds a single glucopyranoside, may have diverged by insertions and deletion of loops in the substrate binding pocket leading to two different kinds of APH enzymes. Notably, the N-lobe and substrate binding regions of NahK are unique and distinct from all other ELK groups. Identification of such family-specific features can aid in the design of substrate-competitive inhibitors.

The study of ELK functional specificity also sheds light on EPK evolution and functions. In particular, substrate specificity in both EPKs and ELKS appears to be mediated through variations at the DFG + 1 position. Previous studies on serine/threonine kinases showed that mutation at the DFG + 1 position shifted phosphor-acceptor specificity between serine and threonine residues [57]. Thus based on these findings, we speculate that substrate specificity in ELKs such as APH2, APH3 and MTRK can be modulated through mutations at the DFG + 1 position. Our study also sheds light on the role of key conserved residues in the active site of protein kinases such as the lysine/arginine (K168^PKA^) in the catalytic loop. The convergent evolution of a lysine in actin kinases in particular indicates that a lysine in the active site is required for protein kinase activity. A quantum mechanical study suggested that this lysine stabilizes a phosphate intermediate during phosphoryl-transfer [50]. Such stabilization may not be required in other small molecule kinases, many of which phosphorylate sugar moieties with more labile hydrogens. Also, the evolution of protein substrate specificity in EPKs appears to have occurred in step-wise fashion, with the addition of specific flexible inserts, such as the activation loop and GHI helices that are unique to EPKs [39]. Likewise, the selective conservation of glycines in the glycine rich loop of EPKs appears to confer flexibility in the ATP binding pocket that is absent in ELKs. Many of these observations would not be possible without an evolutionary model of the entire superfamily that incorporates neo-functionalization. As sequence, structure and functional data on ELKs continues to grow, future efforts will focus on detailed models of ELK neo-functionalization.

## Methods

### Generation of core domain alignment of ELK and EPK groups

Sequences of known EPK and ELK sequences were obtained from Pfam v23.0. [58]. Seed sequences of ELK and EPK groups given in Fig. 2 were obtained from Uniprot [59] using the Pfam identifier of the family as a query, and supplemented with sequences from the annotated genomes of model organisms. A representative PDB structure from each ELK and EPK group was used for structural alignment (PDB ids are given in Fig. 2). Pairwise structural alignments of each ELK and EPK representative PDB structure with Rio kinase (pdbid : 1zp9) were generated using MASS [60], Matt [61] and DeepAlign [62]. Secondary structure elements and Hanks and Hunter subdomain motifs were aligned manually. These structural and motif landmarks ensured correct placement of intervening regions despite the absence of significant sequence similarity. The proteins within each group were aligned against that group’s representative PDB sequence.

MAPGAPS [63], a program to align sequences to a hierarchical set of profiles, was used to generate the final core domain alignment. The input to MAPGAPS is a set of alignment profiles, a consensus sequence for each profile, and a manually-curated template alignment of the consensus sequences. The template alignment defines both the hierarchical relationships between profiles, the alignment of each profile to its parent profile within the hierarchy and, consequently, the alignment of each profile to the root profile, which, in our case, corresponds to the ELK structural core. Based on this input, MAPGAPS identifies those database sequences with a significant match to at least one of the profiles, optimally aligns each matching sequence to its highest-scoring profile and, based on the template alignment, aligns all of the sequences to the ELK structural core. This yields an accurate core alignment by first aligning each database sequence to its most closely-related profile and then aligning each profile alignment to the structural core based on the (manually-curated) template alignment.

More specifically, we iteratively applied the following seven-step procedure:

1. Use each representative PDB sequence both as a master sequence to generate a subgroup profile alignment and as the “consensus” sequence for that subgroup. At this step, phylogenetically weighted consensus generation was done.
2. Use pairwise structure based alignments to generate a template alignment of all PDB sequences. A Rio kinase-anchored template alignment was used as a starting point in the first iteration.
3. Generate a consensus sequences from each profile alignment. At this step, an unweighted consensus master alignment was generated.
4. Generate MAPGAPS profiles from both the template alignment and group alignments.
5. Re-align sequences within each group and generate a consensus sequence; note that this consensus is different from the PDB representative, to which it nevertheless shares high sequence similarity.
6. Align the new consensus sequences using MAPGAPS and the MAPGAPS profiles; this generates a new master alignment that is not Rio anchored.
7. Re-generate MAPGAPS profiles using the new master alignment of consensus sequences as a template and re-aligned group alignments.

### Generation of a maximum likelihood tree of representative sequences

The representative sequences from each ELK group with known structures were taken from the PDB database. For families with no structural information (e.g. Kdo, MalK and RevK) a Uniprot or NCBI sequence was used. The alignment of the sequences of representative structures and Uniprot sequences was done using the MAPGAPS profiles. A maximum likelihood tree with bootstrap support was constructed with RAxML v7.0 [64]. Bootstrap values were estimated with 500 alternate trees generated from the alignment. The ML tree generation used a BLOSUM62 matrix and the consensus tree shown in Fig. 2 was generated using the extended majority rule of RAxML. The tree was colored and visualized using iTOL [65].

### mcBPPS analysis of ELK groups

Residues most characteristic of major ELK groups were identified using the multiple category Bayesian Partitioning with Pattern Selection (mcBPPS) program [66]. Briefly, the mcBPPS program uses Bayesian inference to optimally partition a multiple alignment into predefined subgroups based on those discriminating sequence patterns that most distinguish each subgroup from other subgroups. The input to the program is (1) a master alignment of all the sequences (only core domain was used for mcBPPS) (2) Seed profiles for each subgroup (3) a tree file giving the hierarchy. For the analysis in this manuscript, the tree is given as Additional file 7: Figure S7. In the tree-defining file, the groups marked with “?” are higher level groups such as “ELK”. A single profile was generated for each of the ELK groups and each ELK group was compared in the background of all other ELK groups, excluding EPKs and APKs such as PI3Ks. Input files for running mcBPPS analysis on major ELK groups can be downloaded from: https://bitbucket.org/esbg/elk-mcbpps_input_files.

The determination of most distinguishing sequence patterns requires Markov chain Monte Carlo (MCMC) sampling because, a *priori*, we know neither those sequences assigned to each subgroup, nor the pattern positions for that subgroup, nor the conserved residues defining each pattern. In addition to the input sequence alignment, the mcBPPS program requires a set of predefined, hierarchically arranged subgroups and, for each subgroup, a corresponding “seed alignment” consisting of a few sequences known to belong to that subgroup. The latter helps define the subgroup inasmuch as the corresponding pattern is required to match the consensus for the seed alignment. The mcBPPS program starts with random subgroup assignments for the remaining (non-seed) sequences and with random residue patterns at randomly selected positions. (Note that the residue set defined at each pattern position corresponds to either a single amino acid residue or a small set of biochemically-related amino acid residues.) It then samples over the ‘space’ of possible sequence assignments and patterns for each subgroup based on the following scheme: each node (i.e., subgroup) in the (predefined) hierarchy is defined both a foreground set, consisting of those sequences currently assigned to the subtree rooted at that node, and a background set, consisting of the remaining sequences assigned to the subtree rooted at the parent of that node. During sampling, the mcBPPS program iteratively reassigns sequences and patterns so as to favor a configuration where the subgroup patterns optimally distinguish the foreground from the background sequences. Hence, the mcBPPS sampler is designed to optimally define both the sequences belonging to each subgroup and those conserved residues that most distinguish that subgroup from closely related subgroups. When conserved across evolutionarily distant organisms, these residues are presumably associated with biochemical and structural properties responsible for the corresponding proteins’ subgroup-specific functions.

### Availability of supporting data

All supporting data are included as additional files.

## Additional files

Additional file 1: Figure S1. Tree showing the relationships found between various groups using core domains. **A)** EPK tree of all human kinases showing major groups and their relationships. Each group is given a distinct color. As can be seen from the tree, each major group clusters together with the exception of DYRKs and CMGCs, which are part of the same group. **B)** PI3K tree using representative sequences belonging to each major PI3K sub group. The Inositol binding PI3Ks and protein binding PI3Ks (mTOR, SMG1, ATR and ATM) cluster separately, as expected. (PNG 161 kb) Additional file 2: Figure S2. Phylogeny and taxonomic analysis of ELKs **A)** Full tree showing the relationships found between various ELK groups using core domains. The nodes are colored according to Fig. 2 coloring scheme. pknBs, which are protein kinases, found in bacteria cluster together with other EPKs suggesting that they are EPKs rather than ELKs. The branch points are annotated with bootstrap values (out of 100) in a maximum likelihood tree. **B)** Taxonomic distribution of APH3 families showing the prevalence of APH3 groups in bacteria, fungi and other eukaryotes. The taxonomic classes are colored according to scheme given in the left top corner of the figure. (PNG 1308 kb) Additional file 3: Figure S3. Structural similarities and differences between APH2 and APH3 enzymes **A)** Structural alignment of all APH2 and APH3 enzymes showing that within a group, the structural divergence is low. **B)** Structural alignments of APH2 (pdbid 4dfb, and colored green) and APH3 (pdbid 4fev, colored blue). The overall structural similarity is low, with APH2 having a more elaborate substrate binding region. Shown as insets (below, right) are two divergent regions within the core domain. These regions are subdomain I containing G-rich loop and subdomain IX containing the F-helix. (PNG 505 kb) Additional file 4: Figure S4. A model of Kdo kinase (swissprot identifier: KDKA_PASPI) using Rio kinase (pdbid 1zp9) as a template. The residues that show up as contrastingly conserved are shown as blue sticks. As can be seen from the model, characteristic residues cluster together near the putative substrate binding region. The two arginines within the substrate binding region may bind Kdo similar to the twin-arg motif in MTRK (see Fig. 4). (PNG 208 kb) Additional file 5: Figure S5. Convergent evolution of catalytic loop lysine. Actin kinase is part of the Alpha kinase group and shows a conserved lysine (K1727) near the active site, which is not part of the catalytic loop. Protein kinases such as PKA have a similar lysine (K168) within the catalytic loop. Note the similarity in the geometry of lysine residue despite the conserved lysine in each kinase being present in different regions of the core domain. (PNG 179 kb) Additional file 6: Figure S6. Different ATP binding modes in ELK groups. The catalytic residues are shown superposed and are well aligned. However, the ATP phosphates occupy different orientations in each ELK group. The ATP carbon atoms are colored according to the ELK groups. ATP carbon atoms in PKA are colored in light pink, ATP carbon atoms in ChoK are colored green, ATP carbon atoms in Rio kinase are colored dark blue, ATP carbon atoms in APH3 are colored cyan, ATP carbon atoms in FruK are colored yellow, ATP carbon atoms in MTRK are colored magenta and GTP carbon atoms in APH2 are colored grey. (PNG 396 kb) Additional file 7: Figure S7. The hyperpartitions that are examined in mcBPPS are given in the form of a tree in this figure. The newick format tree is converted into a hyperpartition, which determines the foreground and backgrounds used for determining the most distinguishing residues. For instance, APH2 family is used once as foreground with all ELKs as background, ignoring the EPK and APK groups. Similar analysis is also carried out for other ELK families. Note that as part of the analysis, EPK and PI3K patterns were also generated, but are not discussed. (PNG 42 kb)

## Abbreviations

EPK: eukaryotic protein kinases
ELK: EPK-like kinases
APK: atypical protein kinases
mcBPPS: multiple category bayesian partitioning with pattern selection
APH: aminoglycoside phosphotransferases

## References

[1] Kannan N, Taylor SS, Zhai Y, Venter JC, Manning G (2007). Structural and functional diversity of the microbial kinome. PLoS Biol.

[2] Oruganty K, Kannan N (2012). Design principles underpinning the regulatory diversity of protein kinases. Philos Trans R Soc Lond Ser B Biol Sci.

[3] Hanks SK, Hunter T (1995). Protein kinases 6. The eukaryotic protein kinase superfamily: kinase (catalytic) domain structure and classification. FASEB J.

[4] Hynes NE, Ingham PW, Lim WA, Marshall CJ, Massague J, Pawson T (2013). Signalling change: signal transduction through the decades. Nat Rev Mol Cell Biol.

[5] Pawson T, Scott JD (2005). Protein phosphorylation in signaling--50 years and counting. Trends Biochem Sci.

[6] Schlessinger J. Receptor tyrosine kinases: legacy of the first two decades. Cold Spring Harb Perspect Biol. 2014;6. 10.1101/cshperspect.a008912 PMC3949355 24591517

[7] Thorner J, Hunter T, Cantley LC, Sever R. Signal transduction: from the atomic age to the post-genomic era. Cold Spring Harb Perspect Biol. 2014;6. 10.1101/cshperspect.a022913 PMC4292159 25359498

[8] Burk DL, Hon WC, Leung AK, Berghuis AM (2001). Structural analyses of nucleotide binding to an aminoglycoside phosphotransferase. Biochemistry.

[9] Nurizzo D, Shewry SC, Perlin MH, Brown SA, Dholakia JN, Fuchs RL (2003). The crystal structure of aminoglycoside-3’-phosphotransferase-IIa, an enzyme responsible for antibiotic resistance. J Mol Biol.

[10] Peisach D, Gee P, Kent C, Xu Z (2003). The crystal structure of choline kinase reveals a eukaryotic protein kinase fold. Structure.

[11] Zheng J, Trafny EA, Knighton DR, Xuong NH, Taylor SS, Ten Eyck LF (1993). 2.2 A refined crystal structure of the catalytic subunit of cAMP-dependent protein kinase complexed with MnATP and a peptide inhibitor. Acta Crystallogr D Biol Crystallogr.

[12] Hubbard SR (1997). Crystal structure of the activated insulin receptor tyrosine kinase in complex with peptide substrate and ATP analog. EMBO J.

[13] Davis TL, Walker JR, Allali-Hassani A, Parker SA, Turk BE, Dhe-Paganon S (2009). Structural recognition of an optimized substrate for the ephrin family of receptor tyrosine kinases. FEBS J.

[14] Mah AS, Elia AE, Devgan G, Ptacek J, Schutkowski M, Snyder M (2005). Substrate specificity analysis of protein kinase complex Dbf2-Mob1 by peptide library and proteome array screening. BMC Biochem.

[15] Smith FD, Samelson BK, Scott JD (2011). Discovery of cellular substrates for protein kinase A using a peptide array screening protocol. Biochem J.

[16] Duarte ML, Pena DA, Nunes Ferraz FA, Berti DA, Paschoal Sobreira TJ, Costa-Junior HM (2014). Protein folding creates structure-based, noncontiguous consensus phosphorylation motifs recognized by kinases. Sci Signal.

[17] Creixell P, Palmeri A, Miller CJ, Lou HJ, Santini CC, Nielsen M (2015). Unmasking determinants of specificity in the human kinome. Cell.

[18] Ubersax JA, Ferrell JE (2007). Mechanisms of specificity in protein phosphorylation. Nat Rev Mol Cell Biol.

[19] Bardwell AJ, Frankson E, Bardwell L (2009). Selectivity of docking sites in MAPK kinases. J Biol Chem.

[20] Tokunaga Y, Takeuchi K, Takahashi H, Shimada I (2014). Allosteric enhancement of MAP kinase p38alpha’s activity and substrate selectivity by docking interactions. Nat Struct Mol Biol.

[21] Granum S, Sundvold-Gjerstad V, Gopalakrishnan RP, Berge T, Koll L, Abrahamsen G (2014). The kinase Itk and the adaptor TSAd change the specificity of the kinase Lck in T cells by promoting the phosphorylation of Tyr192. Sci Signal.

[22] Joseph RE, Min L, Xu R, Musselman ED, Andreotti AH (2007). A remote substrate docking mechanism for the tec family tyrosine kinases. Biochemistry.

[23] Hoshi N, Langeberg LK, Scott JD (2005). Distinct enzyme combinations in AKAP signalling complexes permit functional diversity. Nat Cell Biol.

[24] Appel S, Morgan KG (2010). Scaffolding proteins and non-proliferative functions of ERK1/2. Commun Integr Biol.

[25] Gogl G, Schneider KD, Yeh BJ, Alam N, Nguyen Ba AN, Moses AM (2015). The structure of an NDR/LATS kinase-mob complex reveals a novel kinase-coactivator System and substrate docking mechanism. PLoS Biol.

[26] Woegerbauer M, Kuffner M, Domingues S, Nielsen KM (2015). Involvement of aph(3’)-IIa in the formation of mosaic aminoglycoside resistance genes in natural environments. Front Microbiol.

[27] Shi K, Caldwell SJ, Fong DH, Berghuis AM (2013). Prospects for circumventing aminoglycoside kinase mediated antibiotic resistance. Front Cell Infect Microbiol.

[28] Chow JW (2000). Aminoglycoside resistance in enterococci. Clin Infect Dis.

[29] Stogios PJ, Spanogiannopoulos P, Evdokimova E, Egorova O, Shakya T, Todorovic N (2013). Structure-guided optimization of protein kinase inhibitors reverses aminoglycoside antibiotic resistance. Biochem J.

[30] Glunde K, Bhujwalla ZM, Ronen SM (2011). Choline metabolism in malignant transformation. Nat Rev Cancer.

[31] Janardhan S, Srivani P, Sastry GN (2006). Choline kinase: an important target for cancer. Curr Med Chem.

[32] Leonard CJ, Aravind L, Koonin EV (1998). Novel families of putative protein kinases in bacteria and archaea: evolution of the “eukaryotic” protein kinase superfamily. Genet Res.

[33] Krupa A, Srinivasan N (2002). Lipopolysaccharide phosphorylating enzymes encoded in the genomes of Gram-negative bacteria are related to the eukaryotic protein kinases. Protein Sci.

[34] Fraga J, Maranha A, Mendes V, Pereira PJ, Empadinhas N, Macedo-Ribeiro S (2015). Structure of mycobacterial maltokinase, the missing link in the essential GlgE-pathway. Sci Rep.

[35] Scheeff ED, Axelrod HL, Miller MD, Chiu HJ, Deacon AM, Wilson IA (2010). Genomics, evolution, and crystal structure of a new family of bacterial spore kinases. Proteins.

[36] Scheeff ED, Bourne PE (2005). Structural evolution of the protein kinase-like superfamily. PLoS Comput Biol.

[37] Grishin NV (1999). Phosphatidylinositol phosphate kinase: a link between protein kinase and glutathione synthase folds. J Mol Biol.

[38] Yamaguchi H, Matsushita M, Nairn AC, Kuriyan J (2001). Crystal structure of the atypical protein kinase domain of a TRP channel with phosphotransferase activity. Mol Cell.

[39] Kannan N, Neuwald AF (2005). Did protein kinase regulatory mechanisms evolve through elaboration of a simple structural component?. J Mol Biol.

[40] Manning G, Whyte DB, Martinez R, Hunter T, Sudarsanam S (2002). The protein kinase complement of the human genome. Science.

[41] Walker EH, Perisic O, Ried C, Stephens L, Williams RL (1999). Structural insights into phosphoinositide 3-kinase catalysis and signalling. Nature.

[42] Hirsch E, Braccini L, Ciraolo E, Morello F, Perino A (2009). Twice upon a time: PI3K’s secret double life exposed. Trends Biochem Sci.

[43] Tagliabracci VS, Engel JL, Wen J, Wiley SE, Worby CA, Kinch LN, et al. Secreted kinase phosphorylates extracellular proteins that regulate biomineralization. Science. 2012;336:1150–3. 10.1126/science.1217817 PMC3754843 22582013

[44] Ortiz-Lombardia M, Pompeo F, Boitel B, Alzari PM (2003). Crystal structure of the catalytic domain of the PknB serine/threonine kinase from Mycobacterium tuberculosis. J Biol Chem.

[45] Yuan C, Kent C (2004). Identification of critical residues of choline kinase A2 from Caenorhabditis elegans. J Biol Chem.

[46] Ku SY, Cornell KA, Howell PL (2007). Structure of Arabidopsis thaliana 5-methylthioribose kinase reveals a more occluded active site than its bacterial homolog. BMC Struct Biol.

[47] Ku SY, Yip P, Cornell KA, Riscoe MK, Behr JB, Guillerm G (2007). Structures of 5-methylthioribose kinase reveal substrate specificity and unusual mode of nucleotide binding. J Biol Chem.

[48] Heyes DJ, Levy C, Lafite P, Roberts IS, Goldrick M,  Stachulski AV (2009). Structure-based mechanism of CMP-2-keto-3-deoxymanno-octulonic acid synthetase: convergent evolution of a sugar-activating enzyme with DNA/RNA polymerases. J Biol Chem.

[49] Cheng Y, Zhang Y, McCammon JA (2005). How does the cAMP-dependent protein kinase catalyze the phosphorylation reaction: an ab initio QM/MM study. J Am Chem Soc.

[50] Valiev M, Yang J, Adams JA, Taylor SS, Weare JH (2007). Phosphorylation reaction in cAPK protein kinase-free energy quantum mechanical/molecular mechanics simulations. J Phys Chem B.

[51] Zhou B, Wong CF (2009). A computational study of the phosphorylation mechanism of the insulin receptor tyrosine kinase. J Phys Chem B.

[52] Wright GD, Thompson PR (1999). Aminoglycoside phosphotransferases: proteins, structure, and mechanism. Front Biosci.

[53] Daigle DM, McKay GA, Thompson PR, Wright GD (1999). Aminoglycoside antibiotic phosphotransferases are also serine protein kinases. Chem Biol.

[54] Stefely JA, Reidenbach AG, Ulbrich A, Oruganty K, Floyd BJ, Jochem A (2015). Mitochondrial ADCK3 employs an atypical protein kinase-like fold to enable coenzyme Q biosynthesis. Mol Cell.

[55] Smith CA, Toth M, Frase H, Byrnes LJ, Vakulenko SB (2012). Aminoglycoside 2”-phosphotransferase IIIa (APH(2”)-IIIa) prefers GTP over ATP: structural templates for nucleotide recognition in the bacterial aminoglycoside-2” kinases. J Biol Chem.

[56] Wang KC, Lyu SY, Liu YC, Chang CY, Wu CJ, Li TL (2014). Insights into the binding specificity and catalytic mechanism of N-acetylhexosamine 1-phosphate kinases through multiple reaction complexes. Acta Crystallogr D Biol Crystallogr.

[57] Chen C, Ha BH, Thevenin AF, Lou HJ, Zhang R (2014). Identification of a major determinant for serine-threonine kinase phosphoacceptor specificity. Mol Cell.

[58] Finn RD, Bateman A, Clements J, Coggill P, Eberhardt RY (2014). Pfam: the protein families database. Nucleic Acids Res.

[59] UniProt Consortium (2014). UniProt: a hub for protein information. Nucleic Acids Res.

[60] Dror O, Benyamini H, Nussinov R, Wolfson HJ (2003). Multiple structural alignment by secondary structures: algorithm and applications. Protein Sci.

[61] Menke M, Berger B, Cowen L (2008). Matt: local flexibility aids protein multiple structure alignment. PLoS Comput Biol.

[62] Wang S, Ma J, Peng J, Xu J (2013). Protein structure alignment beyond spatial proximity. Sci Rep.

[63] Neuwald AF (2009). Rapid detection, classification and accurate alignment of up to a million or more related protein sequences. Bioinformatics.

[64] Stamatakis A (2006). RAxML-VI-HPC: maximum likelihood-based phylogenetic analyses with thousands of taxa and mixed models. Bioinformatics.

[65] Letunic I, Bork P (2011). Interactive Tree Of Life v2: online annotation and display of phylogenetic trees made easy. Nucleic Acids Res.

[66] Neuwald AF (2011). Surveying the manifold divergence of an entire protein class for statistical clues to underlying biochemical mechanisms. Stat Appl Genet Mol Biol.

