# Supplementary figures and images for: Identification and classification of small molecule kinases: insights into substrate recognition and specificity
Source: BMC Evol Biol. 2016 Jan 6;16:7. doi: 10.1186/s12862-015-0576-x (PMC4702295; doi:10.1186/s12862-015-0576-x)

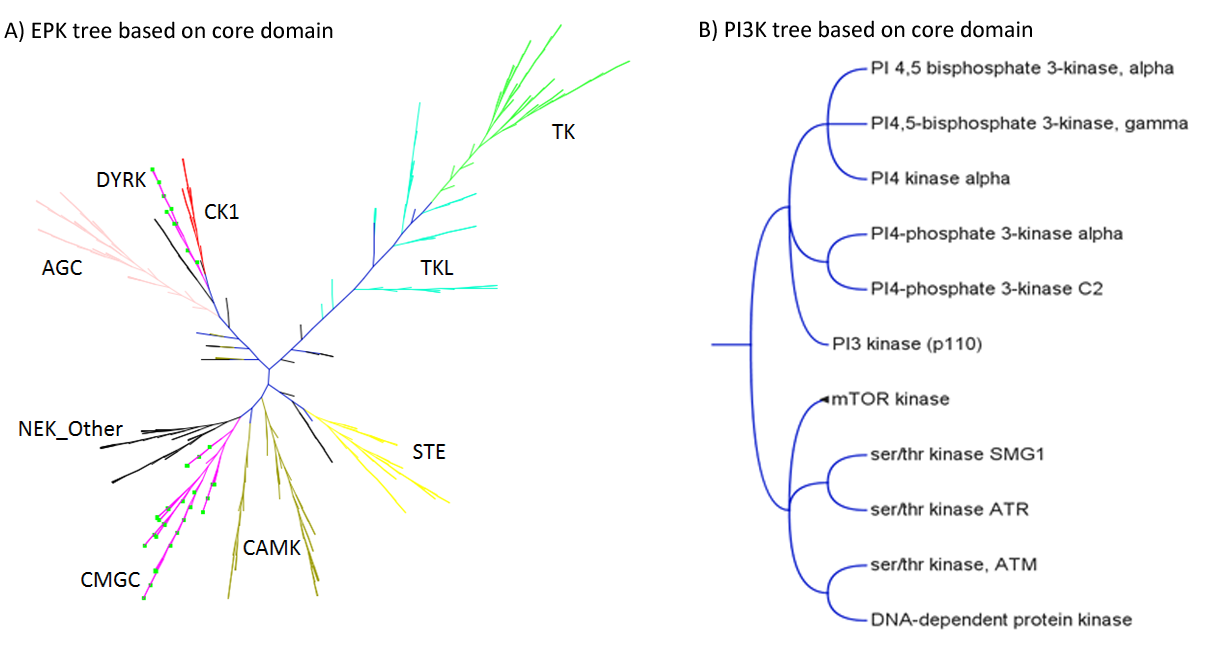

Supplement: Additional file 1: Figure S1. — Tree showing the relationships found between various groups using core domains. A) EPK tree of all human kinases showing major groups and their relationships. Each group is given a distinct color. As can be seen from the tree, each major group clusters together with the exception of DYRKs and CMGCs, which are part of the same group. B) PI3K tree using representative sequences belonging to each major PI3K sub group. The Inositol binding PI3Ks and protein binding PI3Ks (mTOR, SMG1, ATR and ATM) cluster separately, as expected. (PNG 161 kb) [file 12862_2015_576_MOESM1_ESM.png]

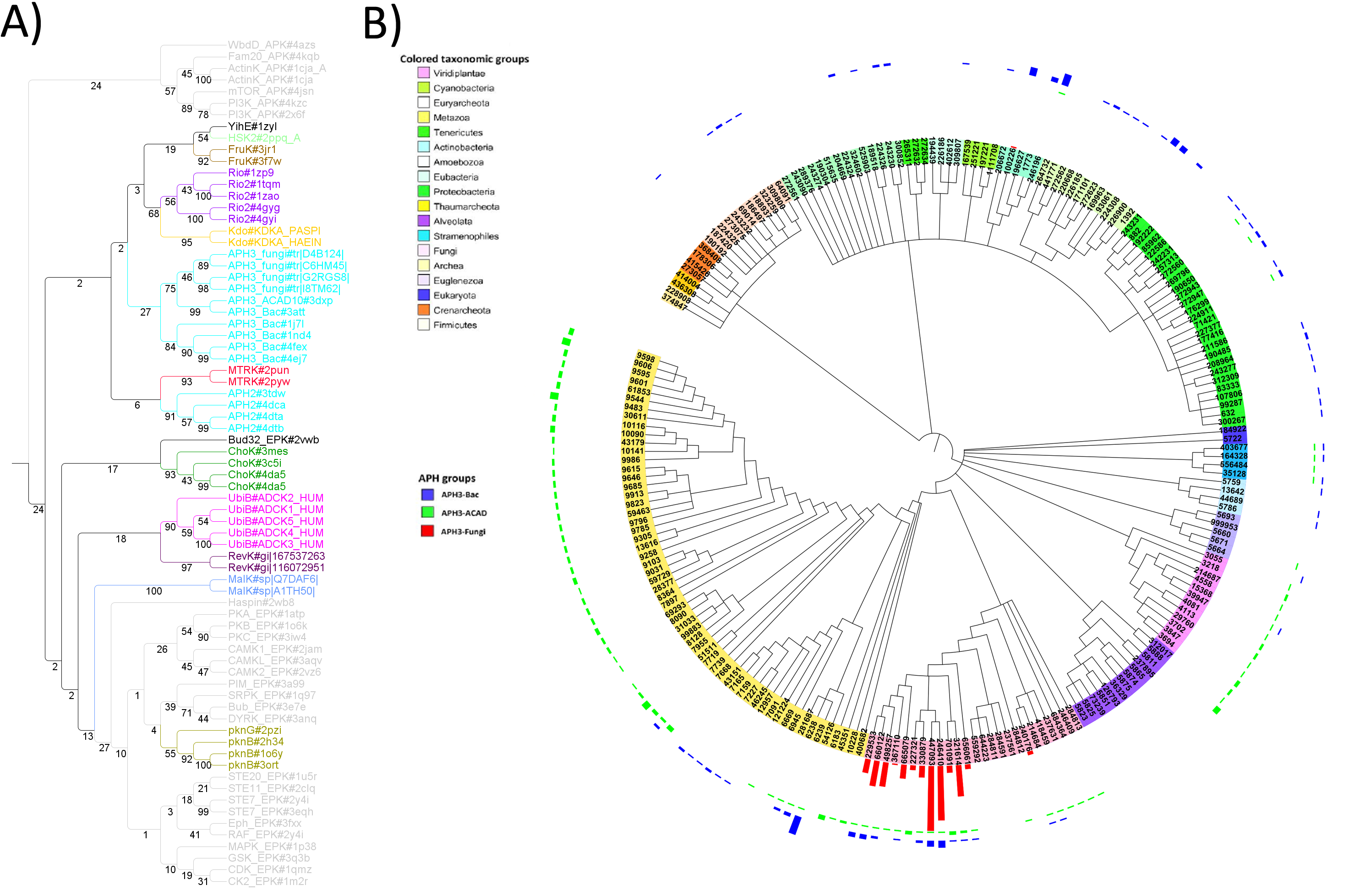

Supplement: Additional file 2: Figure S2. — Phylogeny and taxonomic analysis of ELKs A) Full tree showing the relationships found between various ELK groups using core domains. The nodes are colored according to Fig. 2 coloring scheme. pknBs, which are protein kinases, found in bacteria cluster together with other EPKs suggesting that they are EPKs rather than ELKs. The branch points are annotated with bootstrap values (out of 100) in a maximum likelihood tree. B) Taxonomic distribution of APH3 families showing the prevalence of APH3 groups in bacteria, fungi and other eukaryotes. The taxonomic classes are colored according to scheme given in the left top corner of the figure. (PNG 1308 kb) [file 12862_2015_576_MOESM2_ESM.png]

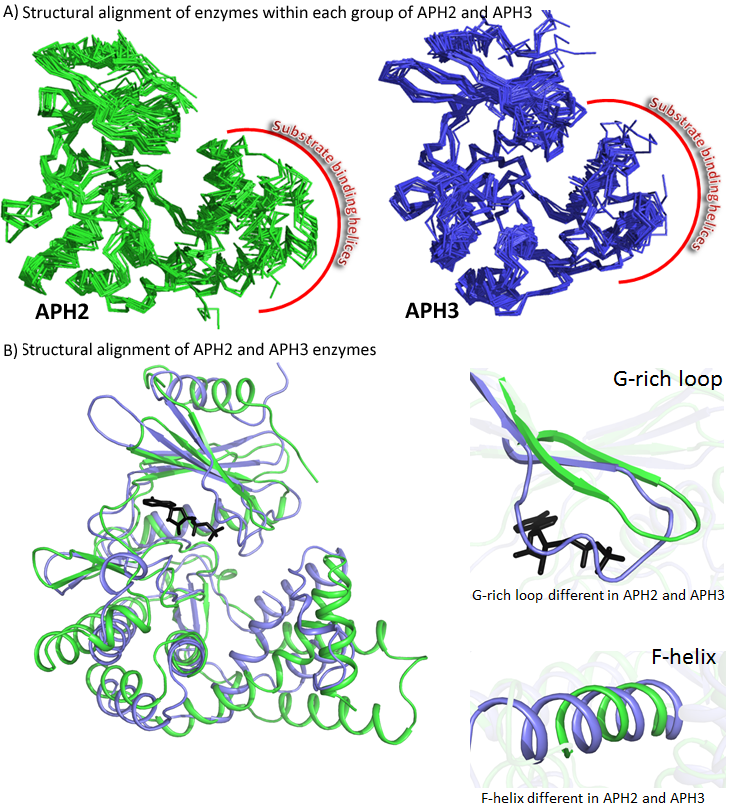

Supplement: Additional file 3: Figure S3. — Structural similarities and differences between APH2 and APH3 enzymes A) Structural alignment of all APH2 and APH3 enzymes showing that within a group, the structural divergence is low. B) Structural alignments of APH2 (pdbid 4dfb, and colored green) and APH3 (pdbid 4fev, colored blue). The overall structural similarity is low, with APH2 having a more elaborate substrate binding region. Shown as insets (below, right) are two divergent regions within the core domain. These regions are subdomain I containing G-rich loop and subdomain IX containing the F-helix. (PNG 505 kb) [file 12862_2015_576_MOESM3_ESM.png]

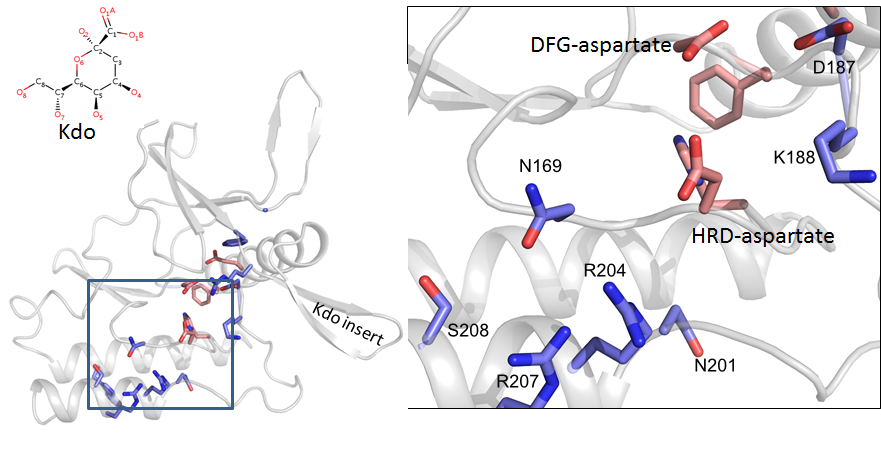

Supplement: Additional file 4: Figure S4. — A model of Kdo kinase (swissprot identifier: KDKA_PASPI) using Rio kinase (pdbid 1zp9) as a template. The residues that show up as contrastingly conserved are shown as blue sticks. As can be seen from the model, characteristic residues cluster together near the putative substrate binding region. The two arginines within the substrate binding region may bind Kdo similar to the twin-arg motif in MTRK (see Fig. 4). (PNG 208 kb) [file 12862_2015_576_MOESM4_ESM.png]

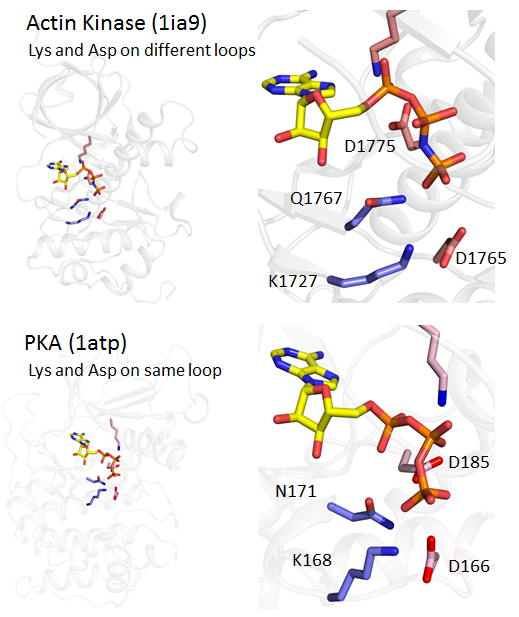

Supplement: Additional file 5: Figure S5. — Convergent evolution of catalytic loop lysine. Actin kinase is part of the Alpha kinase group and shows a conserved lysine (K1727) near the active site, which is not part of the catalytic loop. Protein kinases such as PKA have a similar lysine (K168) within the catalytic loop. Note the similarity in the geometry of lysine residue despite the conserved lysine in each kinase being present in different regions of the core domain. (PNG 179 kb) [file 12862_2015_576_MOESM5_ESM.png]

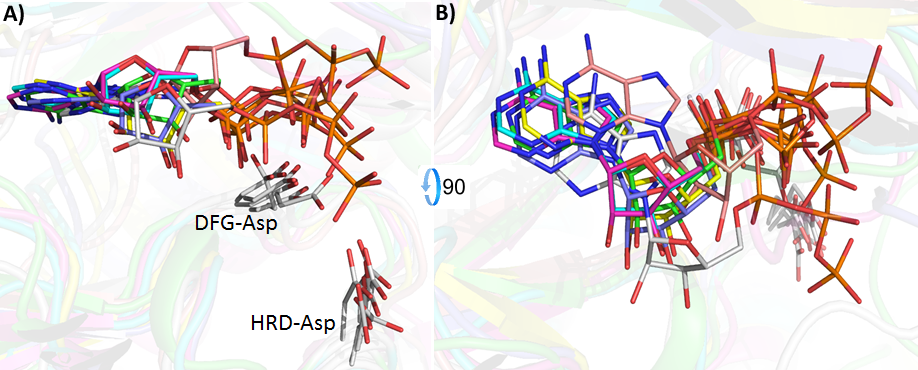

Supplement: Additional file 6: Figure S6. — Different ATP binding modes in ELK groups. The catalytic residues are shown superposed and are well aligned. However, the ATP phosphates occupy different orientations in each ELK group. The ATP carbon atoms are colored according to the ELK groups. ATP carbon atoms in PKA are colored in light pink, ATP carbon atoms in ChoK are colored green, ATP carbon atoms in Rio kinase are colored dark blue, ATP carbon atoms in APH3 are colored cyan, ATP carbon atoms in FruK are colored yellow, ATP carbon atoms in MTRK are colored magenta and GTP carbon atoms in APH2 are colored grey. (PNG 396 kb) [file 12862_2015_576_MOESM6_ESM.png]

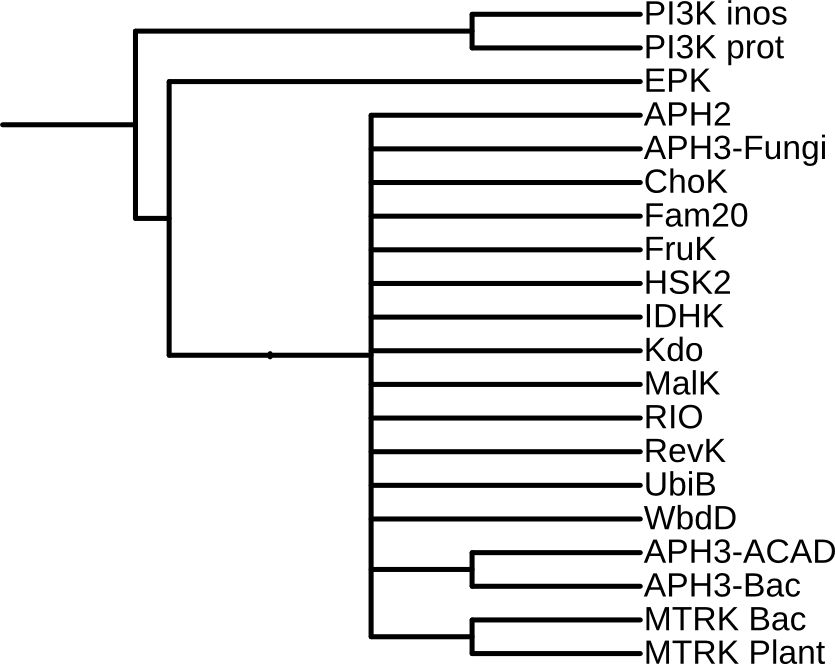

Supplement: Additional file 7: Figure S7. — The hyperpartitions that are examined in mcBPPS are given in the form of a tree in this figure. The newick format tree is converted into a hyperpartition, which determines the foreground and backgrounds used for determining the most distinguishing residues. For instance, APH2 family is used once as foreground with all ELKs as background, ignoring the EPK and APK groups. Similar analysis is also carried out for other ELK families. Note that as part of the analysis, EPK and PI3K patterns were also generated, but are not discussed. (PNG 42 kb) [file 12862_2015_576_MOESM7_ESM.png]
